# Swiss Cheese Loin: A Rare Initial Presentation of Pott’s Spine

**DOI:** 10.7759/cureus.13912

**Published:** 2021-03-15

**Authors:** Sree Subramaniyan, Souradeep Dutta, Ankit Jain, Abhinaya Reddy, Vishnu Prasad Nelamangala Ramakrishnaiah

**Affiliations:** 1 Surgery, Jawaharlal Institute of Postgraduate Medical Education and Research, Puducherry, IND

**Keywords:** pott’s spine, spinal tuberculosis, cutaneous sinus, paraspinal abscess

## Abstract

Spinal tuberculosis (TB) is the most common form of skeletal TB with an exceedingly diverse set of clinical presentations. Most often, there is a slow onset of the disease with patients presenting only with back pain. Although some patients can present later with neurological deficits or with compressive symptoms of an accompanying cold abscess, an initial presentation of a spontaneous cutaneous fistulization of a paraspinal abscess is rare. We present the case of a young boy with such a primary presentation, with no other common symptoms of spinal TB. He was treated with ultrasound-guided percutaneous drainage of the paraspinal abscess and a multidrug anti-tubercular chemotherapy regimen.

## Introduction

Spinal tuberculosis (TB) is the most common form of skeletal TB. Most often, there is a slow onset of the disease with patients presenting only with back pain. Although some patients can present later with neurological deficits or with compressive symptoms of an accompanying cold abscess, an initial presentation in the form of spontaneous cutaneous fistulization of a paraspinal abscess is rare. In this article, we present the case of a young boy with such a primary presentation, with no other common symptoms of spinal TB.

## Case presentation

An 18-year-old boy presented with complaints of on and off low-grade fever and multiple pus discharging ulcers over the right flank for two months. He reported significant weight loss in the last one year, but there was no history of cough, dyspnea, or hemoptysis. There were no complaints of backache or limb weaknesses. He had undergone appendectomy at an outside hospital nine months back. Based on the histopathology report of mesenteric lymph node TB, he was started on anti-tubercular regimen, which he had taken infrequently for six months.

**Figure 1:**
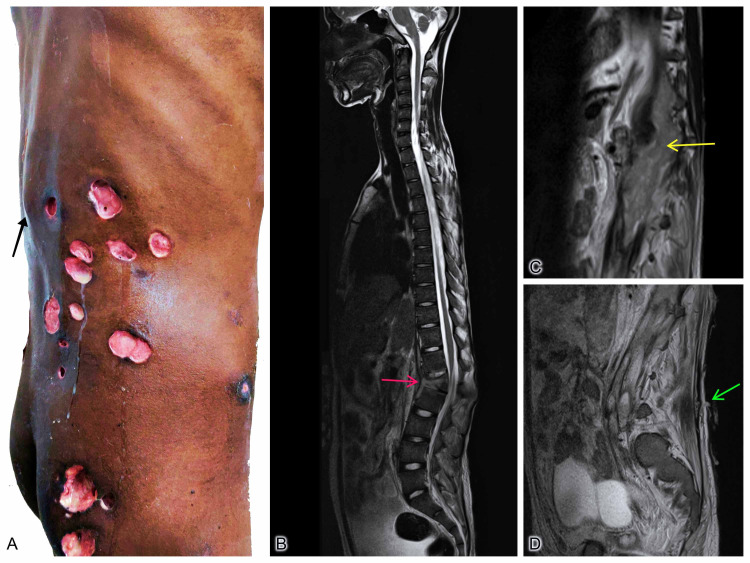
(A) “Swiss Cheese Loin” appearance with multiple ulcers over the right flank and buttock with active pus discharge; (B, C, D) T2 MRI sequences showing the sites of the lesion. Black arrow (A) points to the gibbus deformity at L1 level. Red arrow (B) shows kyphotic angulation due to anterior wedge collapse of L1 vertebral body with normal posterior elements. There is kinking of dura which is abutting the cauda equina nerve roots at this level. Yellow arrow (C) shows abscess collection in the paraspinal region, which is extending into the bilateral psoas regions. Green arrow (D) shows a real-time image of active pus discharge from the cutaneous sinus. MRI, magnetic resonance imaging

On examination, he had multiple active pus discharging ulcers over the right side of the trunk and the right gluteal region (the smallest measuring 2 × 1 × 1 cm; the largest measuring 4 × 4 × 2 cm) (Figure 1A). There was a gibbus deformity with local tenderness on the T12-L1 spine. There was visible fullness in the left paraspinal area. The straight leg raising test caused pain in the lower back. However, neurological examination, including deep tendon reflexes and sensory function, was well preserved. Magnetic resonance imaging (MRI) showed anterior wedge collapse of the L1 vertebral body with a paravertebral collection with subligamentous, epidural, and paravertebral extension and bilateral psoas abscess (Figures 1B, 1C, 1D).

The cartridge-based nucleic acid amplification test (CBNAAT) of the exudate was positive for *Mycobacterium tuberculosis*, sensitive to rifampicin. Ultrasound-guided pigtail catheter drainage of the left paraspinal abscess was done. The four-drug anti-tubercular regimen was restarted, and the patient is now under close follow-up.

## Discussion

Developing countries like India contribute to the major proportion of the global incidence and prevalence of TB. Overall, 10% of all TB cases are extrapulmonary. Skeletal TB constitutes 10-15% of extrapulmonary TB, and one-third of such cases involve the spine [1]. The spread to the spine can be hematogenous, contiguous, or through the lymphatic route. Almost in all cases, the anterior vertebral body is affected first, followed by spread to the paradiscal region (via arterial route) or the central vertebral body (via Batson’s venous plexus). It then spreads under the anterior longitudinal ligament and into the posterior vertebral body, affecting the intervertebral disc at last [1].

Although the Pott’s spine has varied clinical presentation, the most common is back pain [2]. Patients might present with symptoms resulting from the paraspinal cold abscess or the spinal fracture leading to deformity and neurological deficit [3]. Such abscesses containing necrotic debris spread along the paths of least resistance into various tissue planes. The paraspinal cold abscess from the thoracolumbar spine usually present as a swelling in the inferior lumbar triangle and can track down along the iliopsoas muscle to cause pseudo-flexion deformity of the hip. Though there are reports of pus tracking along the femoral or gluteal vessels to present as an abscess in the femoral triangle or gluteal region, respectively [4], spontaneous fistulization of skin in the lumbar area following a Pott’s spine is rare.

MRI helps to ascertain the spinal cord’s status and delineates the soft tissue components (necrotic debris under the anterior and posterior longitudinal ligaments) and activity of the disease by demonstrating disc destruction, vertebral wedging, or marrow edema. The definitive diagnosis depends on identifying the organism using Ziehl-Neelsen stain, CBNAAT techniques, or culture. Multidrug anti-tubercular chemotherapy is the primary modality of treatment. With increasing resistance, antibiotic sensitivity testing is necessary, especially in patients having a recurrent infection or who have received inadequate treatment earlier. Ultrasound-guided percutaneous drainage of a paraspinal tubercular abscess is safe and often the initial treatment modality [5]. Such a drainage with a percutaneous pigtail catheter has the advantage of reducing the cytokine load and preventing further erosion of tissue planes and spread of abscess, promoting quick healing and augmenting the efficacy of the anti-tubercular therapy [6]. The percutaneous catheter can be removed once the drain output becomes minimal, with repeat imaging showing a satisfactory decrease in abscess size. Surgical decompression and fixation are indicated in patients with new-onset or progressive neurological deficits from the spinal cord’s compression [7].

## Conclusions

Although paraspinal abscess is a common sequela of spinal TB, an initial presentation of spontaneous cutaneous sinuses without any neurological deficit is rare. Such a presentation from endemic countries like India warrants a high index of suspicion. Early diagnosis and start of multidrug anti-tuberculosis regimen is the standard of care. Though an uncomplicated paraspinal abscess can often be managed using percutaneous drainage, patients with neurological deficit or spinal deformities require surgical management.
